# Supplementary material for: First field estimation of greenhouse gas release from European soil-dwelling Scarabaeidae larvae targeting the genus Melolontha
Source: PLoS One. 2020 Aug 26;15(8):e0238057. doi: 10.1371/journal.pone.0238057 (PMC7449402; doi:10.1371/journal.pone.0238057)
Supplement: S1 Fig — A test for correlations between paired samples was performed on the entire pooled larval field dataset using Spearman’s rho statistic. Shown are the correlations between CO2 emission (mg h-1 larva-1), CH4 emission (μg h-1 larva-1), larval biomass (g), larval excavation depth (cm below soil surface), air temperature (°C), and incubation time (in minutes). (PDF) [file pone.0238057.s001.pdf]

S1 - Analysis of larval field emissions (on the level of the individual larvae)

A test for correlations between paired samples was performed on the entire pooled larval field dataset using Spearman's rho statistic. The following plot shows the correlations between CO2 emission (mg h-1 larva-1), CH4 emission (µg h-1 larva-1), larval biomass (g), larval excavation depth (cm below soil surface), air temperature (=incubation temperature (°C)), and incubation time (minutes).

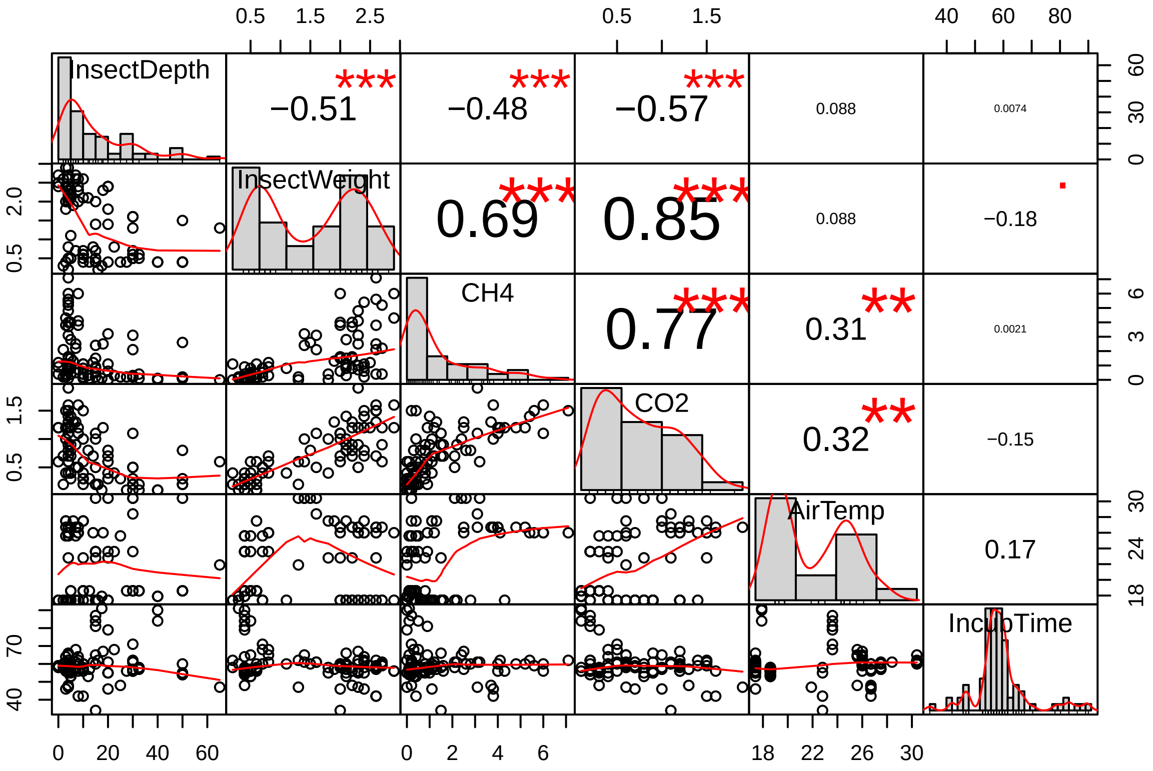

A description of the used R function and the type of plot display can be found at

http://www.sthda.com/english/wiki/correlation-matrix-a-quick-start-guide-to-analyze-format-and-visualize-a-correlation-matrix-using-r-software

(last accessed: 10.05.2020). The following aid to the interpretation of this plot is a citation from this website:

"

- The distribution of each variable is shown on the diagonal.
- On the bottom of the diagonal : the bivariate scatter plots with a fitted line are displayed.
- On the top of the diagonal : the value of the correlation plus the significance level as stars."
