# Supplementary material for: First field estimation of greenhouse gas release from European soil-dwelling Scarabaeidae larvae targeting the genus Melolontha
Source: PLoS One. 2020 Aug 26;15(8):e0238057. doi: 10.1371/journal.pone.0238057 (PMC7449402; doi:10.1371/journal.pone.0238057)
Supplement: Attachment — Submitted filename: Review of First field estimation of greenhouse gas emissions from European soil-dwelling Scarabaeidae larvae targeting the genus Melolontha.pdf [file pone.0238057.s004.pdf]

Merced, California, February 8th, 2020

Dear Dr. Görres and Dr. Kammann,

My name is Angel S. Fernandez-Bou (I prefer to disclose my name to improve transparency of the review process). I have worked for a few years with CO_2 and greenhouse gas emissions from leaf-cutter ant nests in Central America. Our work has followed an environmental engineering approach to measure and model the soil CO_2 dynamics (concentrations and fluxes, and more modestly other greenhouse gases) and their scalability.

I hope my review is useful for you.

Best luck with your research.

Angel S.

Review of “**First field estimation of greenhouse gas emissions from European soil-dwelling Scarabaeidae larvae targeting the genus *Melolontha***”

In this study, the authors incubated beetle larvae in sealed glass tubes to measure their greenhouse gas emissions (mainly CO_2 and CH_4). The focus were the measurements occurred in the field, but the authors also conducted laboratory measurements for comparison purposes. Then, they upscaled the results to provide a more holistic understanding of the impact of those emissions. The main finding (in my opinion) is that laboratory incubations underperformed compared to field measurements, stressing the relevance of conducting field work.

This manuscript is well written in general, and mostly easy to follow for a general educated audience. However, I have a few questions and concerns about the methodology that I hope the authors can clarify. My main concern is that these measurements must be extremely precise, since the GHG emissions of a larva in about one hour are very small. Then, a very minor inconsistency may lead to a major error when upscaled. I also disagree with the wording “CH_4 emissions”, since (as the authors acknowledge) that methane may be consumed in the soil. Therefore, “CH_4 production” sounds more accurate in my opinion.

I have listed (1) the major questions that may require just clarification, but they are key for me to be able to recommend the acceptance of the manuscript; (2) several minor comments and suggestions that are up to the authors to integrate (or not) in their manuscript; and (3) some grammatical comments that I hope can help.

Major questions

Lines 69 and 70. There are several field studies about greenhouse gas emissions from ants in different regions. Certainly not enough, but there are some good examples:

- Roces, Bollacci, Forti, and Kleineidam have done several field studies measuring CO_2 emissions from massive nests in Brazil and South America, even creating casts of the nests.

  Bollazzi M, Forti LC, Roces F (2012) Ventilation of the giant nests of *Atta* leaf-cutting ants: does underground circulating air enter the fungus chambers? Insect. Soc 59:487–498. https://doi.org/10.1007/s00040-012-0243-9

  Kleineidam C, Roces F (2000) Carbon dioxide concentrations and nest ventilation in nests of the leaf-cutting ant *Atta vollenweideri*. Insect Soc 47:241–248. https://doi.org/10.1007/PL00001710

  Kleineidam C, Ernst R, Roces F (2001) Wind-induced ventilation of the giant nests of the leaf-cutting ant *Atta vollenweideri*. Naturwis-senschaften 88:301–305. https://doi.org/10.1007/s001140100235

- Fernandez-Bou, Harmon, and Dierick have two field studies about CO_2 emissions and soil CO_2 dynamics related to leaf-cutter ants that were upscaled to the ecosystem scale in Central America.

  Fernandez-Bou, A. S., Dierick, D., Swanson, A. C., Allen, M. F., Alvarado, A. G. F., Artavia-León, A., et al. (2019). The role of the ecosystem engineer, the leaf-cutter ant *Atta cephalotes*, on soil CO2 dynamics in a wet tropical rainforest. Journal of Geophysical Research: Biogeosciences, 124, 260– 273. https://doi.org/10.1029/2018JG004723

  Fernandez-Bou, A.S., Dierick, D. & Harmon, T.C. Oecologia (2020). Diel pattern driven by free convection controls leaf-cutter ant nest ventilation and greenhouse gas emissions in a Neotropical rain forest. https://doi.org/10.1007/s00442-020-04602-2

- Soper found remarkable N_2O emissions from refuse piles of *Atta colombica* in Costa Rica;

  Soper FM, Sullivan BW, Osborne BB et al (2019) Leaf-cutter ants engineer large nitrous oxide hot spots in tropical forests. Proc R Soc B Biol Sci 286:20182504. https://doi.org/10.1098/rspb.2018.2504

- Emissions from smaller ant nest in northern Europe have been studied by Finér, Risch, Ohashi; Ohashi also has at least a publication with emissions from ants in Thailand.

  Ohashi M, Finér L, Domisch T et al (2005) CO2 efflux from a red wood ant mound in a boreal forest. Agric For Meteorol 130:131–136. https://doi.org/10.1016/j.agrformet.2005.03.002

  Risch A, Schuetz M, Jurgensen MF et al (2005) CO2 emissions from red wood ant (*Formica rufa* group) mounds: seasonal and diurnal patterns related to air temperature. Ann Zool Fenn 42:283–290. https://www.jstor.org/stable/23735916

Lines 123 to 130. Were the vacutainers filled at the same moment than the larvae were placed in the containers for incubation? In other words, were the vacutainers filled with air containing the exact same GHG concentrations that what the larvae containers had in the beginning of the incubation period? This is how the authors calculate the GHG production by the larvae, and if the initial concentrations were not the same, the production would not be reliable. If this is the case, I suggest they rephrase it to something like "…ambient air at the beginning of the field incubations.". This is a major concern for me, since I do not understand why some N_2O flux measurements are negative.

Ln 124 to 125. At what depth were the larvae incubated? Were they incubated at the depth they were found? And did the authors measure the temperature at that depth? It occurs to me that, since temperature at different depths is usually different, changing the temperature at which the larvae were found may have changed their regular metabolism.

Lines 167 to 175. If I understood correctly, you used correlation between pairs of variables. Why? I would probably have used a generalized linear mixed model (glmmPQL, MASS package in R), since some variables can influence and be influenced by others, and interactions among them should be considered. In addition, the plot seems to be a random factor, since there are characteristics intrinsic of the plot for which the measured variables do not account (I like the example of the researcher who asks questions to people; fixed factors are sex, age, education, etc., while the random factor is the person identification, since they have their own personal background that cannot be measured by the fixed factors but influences their response to some extent). I do not mean that GLMM are the only way to do it, but I would like to understand a solid reason why you chose to conduct the correlation analysis.

Also, if you are not familiar with GLMM and are curious, there is a nice manual by Bodo Winter about LMM (https://arxiv.org/ftp/arxiv/papers/1308/1308.5499.pdf).

Lines 178 to 189. Similarly to the comment before, why did you do a simple linear regression considering only one independent variable?

Upscaling. I would have measured the CO_2 and CH_4 flux in the field before excavating the plots. This is a major part of the methodology that I think is missing here, in particular to solidify the possible CH_4 emission claims. As the authors point out, increased CH_4 production leads to the possibility of increased soil CH_4 consumption. And I think the authors agree with this in the conclusions, when they say, "Scarabaeidae larvae should not be neglected as sources of CO_2, CH_4, and N_2O in soil GHG flux research.". They did not measure soil GHG flux in this study and that would have made a more solid study in my opinion, but I do not think that is a reason to reject this manuscript.

The authors discuss and acknowledge the importance of "the effect" of CH_4 production by soil fauna instead of the faunal CH_4 emissions. And to be consistent, in the upscaling results, I think it is more correct to speak about CH_4 production and not about CH_4 emissions, since that CH_4 may not reach the atmosphere as it may be consumed before (therefore, not emitted). This may affect the emissions claims presented in the study and even the title.

Lines 322 and 333. I disagree. The nature of CH_4 emissions by termites is completely different than the potential (if any) soil CH_4 emissions by beetles. As stated before and by the authors, CH_4 produced in the soil matrix is normally consumed as it diffuses through a tortuous and long path, while CH_4 produced in the guts of the termites and by their nest metabolism is connected with the atmosphere by the cathedral vents and transported by convection, a mechanism several orders of magnitude more efficient that soil GHG diffusion. Then, CH_4 produced by termites finds (quite fast) its way to the atmosphere, while that produced by Scarabaeidae likely not.

Minor comments, suggestions, and questions

Line 96. I think it would be useful to mention regular size and weight of the larvae, for those readers not familiar with them.

Lines 109 to 120. Did the authors collect all the larvae present in the plots to account for abundance/density? Is this how they were able to upscale? If yes, I suggest clarifying it in the "Soil excavations" section.

Line 179. What is (or how are the calculations for) the larval density per unit area?

Line 184. What is the literature? Can you add citations and mention the numbers here?

Lines 195 and 196. Here I miss the interaction among variables. For example, does the temperature influence larval biomass, hence indirectly CO_2 production?

Line 200. I disagree with using "CH_4 emissions"; larval "CH_4 production" is more accurate.

Line 223. Larval abundance methods are not clearly explained in the methodology.

Line 232. References?

Line 234. What is the CO_2 efflux por unit area? And CH_4 results should refer to production and not emissions.

Line 234. "in this region" is confusing, since the authors are referring to the European level. Where in Europe (approximately) are they talking?

Line 245 to 246. That is interesting! Also good point in lines 268 and 269.

Line 250 to 252. Why do the authors think only some larvae produce N_2O? Can they add that in the discussion?

Lines 274 to 282. I think this should be revised in line with all the suggestions and comments.

Lines 289 to 307. Very good discussion.

Lines 308 to 314 and S1_File. Why the authors think N_2O flux is negative in a few occasions?

Line 310. Not "considerable," but for ants there is some literature about N_2O (Soper et al, 2019, etc.).

S1_File. The authors do not report the time of sampling, but I wonder if they would have found a diel pattern, for example, consistent with the soil CO_2 diel pattern: when CO_2 concentration in the soil is higher, do the larvae produce the same or less, since there is less O_2?

Grammar and standardization

I kindly suggest that the authors revise the manuscript for consistency in at least three points that I noticed:

1. The authors did not use the serial comma in some occasions, but they did in others. I suggest homogenizing the whole document using the serial comma. For example, in lines 22, 57, 58, 60, etc.

2. A comma should not separate the subject and the verb. For example, in line 20, I believe that the correct way to write this is "...from laboratory experiments is often controversial, and it has been limited...". In lines 48 and 49 "... (N_2O), but they are also...". Other examples of missing subject after comma are in lines 64, 65, 133, etc.

3. I suggest following the International System units and rules. A checklist can be found online (I use this one https://physics.nist.gov/cuu/Units/checklist.html). For example, in lines 31 and 32, units must be provided after the amounts, and I would use "Gg" (giga grams) instead of "kt" (unless the authors think those units are better for broader communication): "10.42 Gg CO_2 yr^{-1} and 409.53 Gg CO_2 yr^{-1}, and 0.01 Gg CH_4 yr^{-1} and 1.36 Gg CH_4 yr^{-1}.". The same occurs in line 74, 88, 89, 99, 100, 149, 150, 163, etc. In line 104, there should be a space character in "1 m". In line 109, I believe the authors want to use "×" (Alt + 0215) instead of "x". In lines 127, 128, and 132, change "ml" to "mL". Line 146, use "Pa" or "kPa" instead of "PSI". And there may be other excerpts needing standardization that the authors will likely find quickly with the help of the checklist.
