# Supplementary material for: First field estimation of greenhouse gas release from European soil-dwelling Scarabaeidae larvae targeting the genus Melolontha
Source: PLoS One. 2020 Aug 26;15(8):e0238057. doi: 10.1371/journal.pone.0238057 (PMC7449402; doi:10.1371/journal.pone.0238057)
Supplement: Attachment — Submitted filename: Comments to Authors.pdf [file pone.0238057.s006.pdf]

Comments to the Authors

First field estimation of greenhouse gas emissions release from 2 European soil-dwelling Scarabaeidae larvae targeting the genus *Melolontha*

The manuscript is well written and has concise, well connected sentences. In this study, the authors have made an attempt to do field and lab scale measurement of greenhouse gas emissions from Scarabaeidae larvae. I really liked the idea of upscaling the results in order to provide better understanding of the impacts of such emissions. Several studies talk about microbial decomposition of soil organic carbon and how that influences the atmospheric greenhouse gas concentrations but very few have talked about other faunal groups such as ants, termites and beetle larva. So, this is a good attempt to quantify that. I have gone through the responses to reviewers' comments and most of them seem satisfactory to me. Some of my overall concerns on the methodology of this manuscript are below.

The larvae were incubated at the soil surface in field, but the larvae were actually excavated from deeper depths. Do you think by doing so, we are adding a bias to the greenhouse gas emissions that actually comes from larvae at deeper depths versus what is reported in the manuscript?

How the calculations for of these emissions were done?

Also, the larvae used in field study and lab incubation, were they all similar in size? If not, do you think that would influence the greenhouse gas emissions as well?

Other than these minor concerns, I have gone through the responses to the reviewers and they seem convincing and satisfactory to me.
