# Supplementary material for: First field estimation of greenhouse gas release from European soil-dwelling Scarabaeidae larvae targeting the genus Melolontha
Source: PLoS One. 2020 Aug 26;15(8):e0238057. doi: 10.1371/journal.pone.0238057 (PMC7449402; doi:10.1371/journal.pone.0238057)
Supplement: Attachment — Submitted filename: Response to Reviewers.docx [file pone.0238057.s005.docx]

**Author Reply**

**First field estimation of greenhouse gas emissions from European soil-dwelling Scarabaeidae larvae targeting the genus Melolontha**

Authors: Carolyn-Monika Görres^1,2,*^, Claudia Kammann^1^

^1^ Department of Applied Ecology, Hochschule Geisenheim University, Geisenheim, Germany

^2^ Department of Soil Science and Plant Nutrition, Hochschule Geisenheim University, Geisenheim, Germany

**Point-by-point reply to Reviewer #1**

*In this study, the authors incubated beetle larvae in sealed glass tubes to measure their greenhouse gas emissions (mainly CO2 and CH4). The focus were the measurements occurred in the field, but the authors also conducted laboratory measurements for comparison purposes. Then, they upscaled the results to provide a more holistic understanding of the impact of those emissions. The main finding (in my opinion) is that laboratory incubations underperformed compared to field measurements, stressing the relevance of conducting field work.*

*This manuscript is well written in general, and mostly easy to follow for a general educated audience. However, I have a few questions and concerns about the methodology that I hope the authors can clarify.*

REPLY: We would like to thank the reviewer for the thorough evaluation of our manuscript and the constructive criticism. We also appreciate his transparency. We hope, we could dispel the reviewer’s concerns with our revision.

*My main concern is that these measurements must be extremely precise, since the GHG emissions of a larva in about one hour are very small. Then, a very minor inconsistency may lead to a major error when upscaled.*

REPLY: The GHG emissions of a larva are actually not that small. Gas chromatographic analysis of the samples is sufficient and the measurements do not have to be extremely precise. We encountered rather the opposite problem that larval emissions were in several cases much higher than expected. We agree with your concern regarding error increase through upscaling. Thus, we find it important to include error propagation in the data analysis and to transparently report such errors. We have added a statistical summary of the gas concentrations measured in the incubation vials to the method section and also included the sample gas concentrations for each individual larva in the supporting information.

*I also disagree with the wording “CH4 emissions”, since (as the authors acknowledge) that methane may be consumed in the soil. Therefore, “CH4 production” sounds more accurate in my opinion.*

REPLY: We understand the issue raised by the reviewer. Depending on one’s viewpoint the word ‘emission’ might be interpreted in a different way than anticipated by us. In soil greenhouse gas flux research, there are two definitions of emission in use. The first is the one you are referring to and one can use for example the UNFCCC Article 1.4 as reference: ‘ “Emissions” means the release of greenhouse gases and/or their precursors into the atmosphere over a specified area and period of time’. However, “emission” can also be used in a much broader sense as simply “the act of sending out gas, heat, light, etc.” (Cambridge Dictionary). In soil fauna literature, the term “emission” is commonly used in its broader definition and the words “emission”, “production”, and also “release” are used interchangeably for gases directly emitted by animals, even within the same sentence (see e.g. Egert et al. 2005 or Ihssen et al. 2003). It is linguistically correct to say that larvae emit CH_4_ into the surrounding soil (or an incubation vial) and we use the term in this broader sense in our manuscript. We don’t think the term “production” is more accurate because one could also question if the gas is really produced the moment it is released from the animal. However, to avoid confusion and to take into account readers from both viewpoints we have modified the manuscript. First, we avoided the term emission in the title, abstract, introduction and conclusion since these are the sections most often just skimmed by readers. Second, we added a term definition to the method section clarifying what we mean by emission when we use it in the methods, results and discussion sections.

*I have listed (1) the major questions that may require just clarification, but they are key for me to be able to recommend the acceptance of the manuscript; (2) several minor comments and suggestions that are up to the authors to integrate (or not) in their manuscript; and (3) some grammatical and standardization comments that I hope can help.*

REPLY:

*Major questions*

*Lines 69 and 70. There are several field studies about greenhouse gas emissions from ants in different regions. Certainly not enough, but there are some good examples:*

*- Roces, Bollacci, Forti, and Kleineidam have done several field studies measuring CO2 emissions from massive nests in Brazil and South America, even creating casts of the nests.*

*- Fernandez-Bou, Harmon, and Dierick have two field studies about CO2 emissions and soil CO2 dynamics related to leaf-cutter ants that were upscaled to the ecosystem scale in Central America.*

*- Soper found remarkable N2O emissions from refuse piles of Atta colombica in Costa Rica;*

*- Emissions from smaller ant nest in northern Europe have been studied by Finér, Risch, Ohashi; Ohashi also has at least a publication with emissions from ants in Thailand.*

REPLY: Thank you for pointing out these studies. We were not aware of this research conducted on ants. However, in the section of the manuscript you are referring to we wanted to lay the focus only on arthropods which are capable of producing CH_4_. We have rephrased the sentences to make this clearer. But we have included some of the ant references in lines 55ff., where we think they fit in better.

*Lines 123 to 130. Were the vacutainers filled at the same moment than the larvae were placed in the containers for incubation? In other words, were the vacutainers filled with air containing the exact same GHG concentrations that what the larvae containers had in the beginning of the incubation period? This is how the authors calculate the GHG production by the larvae, and if the initial concentrations were not the same, the production would not be reliable. If this is the case, I suggest they rephrase it to something like “…ambient air at the beginning of the field incubations.”. This is a major concern for me, since I do not understand why some N2O flux measurements are negative.*

REPLY: All vacutainers had been evacuated in the laboratory prior to the field trip. At the beginning of each incubation, all glass tubes, including the ones for the blank measurement, were waved in the air to assure equal ambient GHG concentrations in all glass tubes at the incubation start. Control vacutainers were filled with ambient air also at this step. So, this is a yes to your first two questions. The blanks were later compared to these air samples to make sure that no additional GHG were released by the used rubber stoppers. We modified the text to make this clearer.

Regarding the negative N_2_O fluxes, the associated error term is always much larger than the flux itself. These fluxes are not differentiable from a zero flux and cannot be considered as truly negative. Some researchers set such flux estimates to zero in a dataset. We decided to leave the original estimates. Which convention with respect to zero fluxes is applied becomes an important decision for flux modelling and upscaling, but since we did not use the N_2_O fluxes for modelling, the decision which convention to use is not of great importance. Significant negative fluxes, regardless of the gas species, would have been a major concern as they might have indicated gas uptake by the rubber stopper. Cases of this happening have been reported in the gas flux community.

*Ln 124 to 125. At what depth were the larvae incubated? Were they incubated at the depth they were found? And did the authors measure the temperature at that depth? It occurs to me that, since temperature at different depths is usually different, changing the temperature at which the larvae were found may have changed their regular metabolism.*

REPLY: Temperatures were only measured at the soil surface before the excavation of a plot and at the bottom of a plot at the excavation end. Excavated larvae were extracted from the plot and were incubated on the soil surface, i.e. the glass tubes were placed onto the soil surface were the temperatures were higher than in the soil. It is definitely possible that this affected the larval metabolism. The excavation itself also presents a stress factor. How significantly this stress affects the GHG emissions is currently unknown, but a question which has to be asked about this incubation method in general. We modified the manuscript to take this into account.

*Lines 167 to 175. If I understood correctly, you used correlation between pairs of variables. Why? I would probably have used a generalized linear mixed model (glmmPQL, MASS package in R), since some variables can influence and be influenced by others, and interactions among them should be considered. In addition, the plot seems to be a random factor, since there are characteristics intrinsic of the plot for which the measured variables do not account (I like the example of the researcher who asks questions to people; fixed factors are sex, age, education, etc., while the random factor is the person identification, since they have their own personal background that cannot be measured by the fixed factors but influences their response to some extent). I do not mean that GLMM are the only way to do it, but I would like to understand a solid reason why you chose to conduct the correlation analysis.*

*Also, if you are not familiar with GLMM and are curious, there is a nice manual by Bodo Winter about LMM (https://arxiv.org/ftp/arxiv/papers/1308/1308.5499.pdf).*

REPLY: The study presented was an explorative pilot field study (we added this to the introduction) with the aim to cover as many different environmental conditions for cockchafers as possible, thus increasing the variability in the dataset. Due to the involved logistics (e.g. site selection and access, cockchafer abundance, soil excavation conditions) this led to a highly unbalanced dataset which complicates the use of advanced statistics. Any data analysis should start descriptively to provide an overview of the data and to uncover first patterns. Especially for such a dataset and study as we have here, we think it is a very important analysis. The question for any analysis should always be what insights can we gain. We tested models with different combinations of insect depth, biomass, location (fixed effect) and species (fixed effect), but gained no further insights. We added a notion to the manuscript that we considered linear mixed models. Yes, plot can be considered as random factor, but accounting for that mainly effects the variance estimates and not the main estimates. This would have been very important if the models were used for upscaling which they were not. With linear mixed models, one can take a closer look at the effects within and between groups, but that was not really the aim of this study. We were just interested in general trends and patterns. Thus, our decision to deal with the hierarchical structure of our data by using the simple approach of data aggregation, fully aware that this approach does not take into account all the potential information contained in the dataset.

Thank you for the link to the GLMM manual.

*Lines 178 to 189. Similarly to the comment before, why did you do a simple linear regression considering only one independent variable?*

REPLY: Please see the previous comment. It performed the best among the different models we tested. An R²_adj_. of over 90 is extremely good for ecological data. Inclusion of any additional variables and/or interaction terms would have only led to marginal improvements. As pointed out in the manuscript, biomass is one variable which integrates many larval traits and also environmental conditions. As such it is not surprising that again and again it emerges as one of the main variables for upscaling emissions. A more complicated model could certainly further improve the fit, but it also increases the risk of overfitting. And from an upscaling perspective, the simpler the model the better. We can only upscale if we have datasets available for each variable on the target scale.

*Upscaling. I would have measured the CO2 and CH4 flux in the field before excavating the plots. This is a major part of the methodology that I think is missing here, in particular to solidify the possible CH4 emission claims. As the authors point out, increased CH4 production leads to the possibility of increased soil CH4 consumption. And I think the authors agree with this in the conclusions, when they say, “Scarabaeidae larvae should not be neglected as sources of CO2, CH4, and N2O in soil GHG flux research.”. They did not measure soil GHG flux in this study and that would have made a more solid study in my opinion, but I do not think that is a reason to reject this manuscript.*

*The authors discuss and acknowledge the importance of “the effect” of CH4 production by soil fauna instead of the faunal CH4 emissions. And to be consistent, in the upscaling results, I think it is more correct to speak about CH4 production and not about CH4 emissions, since that CH4 may not reach the atmosphere as it may be consumed before (therefore, not emitted). This may affect the emissions claims presented in the study and even the title.*

REPLY: We think, the reviewer’s concern stems from the fact that we use the term “emission” in a broader sense than the reviewer (see previous reply regarding the definition of emission). Measuring soil net GHG fluxes was not the aim of this study. It was included in the original field design, but it turned out pretty quickly that it was logistically not feasible. The measurements per plot were so time intensive and laborious that it would have resulted in a drastic reduction of the overall number of measured plots in the dataset. Thus, we focused only on incubating as many larvae as possible. Net soil fluxes will be the next step of our research. The obvious method of choice are chambers, but there are still methodological problems associated with this method when applying it to Scarabaeidae larval studies. Chamber bases installed in the soil can severely restrict the horizontal movement of larvae which might affect biomass consumption as well as larval social behaviour (they are cannibalistic), and thus larval gas release. The chamber method has to be modified first for this type of studies to ensure reliable net flux estimates.

*Lines 322 and 333. I disagree. The nature of CH4 emissions by termites is completely different than the potential (if any) soil CH4 emissions by beetles. As stated before and by the authors, CH4 produced in the soil matrix is normally consumed as it diffuses through a tortuous and long path, while CH4 produced in the guts of the termites and by their nest metabolism is connected with the atmosphere by the cathedral vents and transported by convection, a mechanism several orders of magnitude more efficient that soil GHG diffusion. Then, CH4 produced by termites finds (quite fast) its way to the atmosphere, while that produced by Scarabaeidae likely not.*

REPLY: We want to challenge that statement and point out several things:

- GHG emission research on termites has focused on mound-building termites because they are much easier to find and to study than subterranean and wood-nesting termites. For non-mound building termites, there are no cathedral vents that can channel emissions quickly to the atmosphere. Especially the nature of CH_4_ emissions from subterranean termites should be similar to that from subterranean Scarabaeidae larvae regarding gas transport through the soil matrix.
- Nauer et al. (2018) have demonstrated that microbial CH_4_ oxidation in termite mound material can be substantial and mitigate 20 – 80 % of termite CH_4_ emissions.
- It is known from peatland research that an aerobic zone of 20 cm between the soil surface and the groundwater table is often sufficient for complete microbial oxidation of CH_4_ produced in the waterlogged soil zone. However, if we look for example at *Melolontha melolontha*, they feed right below the turf, leaving less than 5 cm of soil depth for microbial CH_4_ oxidation. Is such a shallow soil layer really sufficient to mitigate all CH_4_ released from the larvae? We don’t know, but it might not be. For forests, there is increasing research evidence on CH_4_ transmitted via trees to the atmosphere. If *Melolontha* *hippocastani* larvae emit CH_4_ while feeding on tree roots, could these emissions be channelled through the tree roots and stems into the atmosphere, bypassing soil methane oxidizers? Again, we don’t know, but it might be possible.

We added some of these aspects to the discussion.

*Minor comments, suggestions, and questions*

*Line 96. I think it would be useful to mention regular size and weight of the larvae, for those readers not familiar with them.*

REPLY: We added a reference for larval size, but we couldn’t find a reference yet for weight.

*Lines 109 to 120. Did the authors collect all the larvae present in the plots to account for abundance/density? Is this how they were able to upscale? If yes, I suggest clarifying it in the “Soil excavations” section.*

REPLY: Yes, all larvae in a plot were collected. And then we just multiplied the total number of larvae by 4 since a plot covered an area of 0.25 m². We added that to the manuscript.

*Line 179. What is (or how are the calculations for) the larval density per unit area?*

REPLY: See previous reply. The calculation was exactly the same as for biomass and emissions. We added that to the soil excavations section.

*Line 184. What is the literature? Can you add citations and mention the numbers here?*

REPLY: Done.

*Lines 195 and 196. Here I miss the interaction among variables. For example, does the temperature influence larval biomass, hence indirectly CO2 production?*

REPLY: We did not see a direct short-term interaction between temperature and biomass. This makes sense as for example the same biomass can be encountered at different temperatures. The biomass of a second instar larva in autumn before the winter rest will coincide with high soil temperatures, while the same larva measured during spring emergence will be encountered at much lower soil temperatures, but biomass has not increased during the winter time. The effect of temperature on larval biomass has to be viewed more on a seasonal scale. Higher temperatures can increase larval growth and shorten the entire life cycle by a year, while in vegetation periods with colder than normal temperatures, larval growth can be significantly slowed down.

We saw a weak correlation between gaseous carbon production and incubation temperature and added that to the text.

*Line 200. I disagree with using “CH4 emissions”; larval “CH4 production” is more accurate.*

REPLY: See our previous comments on that topic.

*Line 223. Larval abundance methods are not clearly explained in the methodology.*

REPLY: Corrected. See previous comments.

*Line 232. References?*

REPLY: We added the reference to the method section.

*Line 234. What is the CO2 efflux por unit area? And CH4 results should refer to production and not emissions.*

REPLY: Please see our previous replies regarding the term “emission”. The efflux is for the entire 200,000 ha combined. The CO2 efflux per unit area are displayed in Fig. 2.

*Line 234. "in this region" is confusing, since the authors are referring to the European level. Where in Europe (approximately) are they talking?*

REPLY: This refers to the 200,000 ha. We rephrased the sentence. The European countries which are included in this 200,000 ha estimate have been added to the discussion section.

*Line 245 to 246. That is interesting! Also good point in lines 268 and 269.*

REPLY: Thank you.

*Line 250 to 252. Why do the authors think only some larvae produce N2O? Can they add that in the discussion?*

REPLY: It is known for earthworms that their don’t have N_2_O-producing microorganisms endemically living in their gut system. Instead, ingested soil denitrifiers are activated during their gut passage. Maybe it is the same principle in cockchafer larvae and their N_2_O emissions depend on ingested soil microorganisms. Since *Melolontha* spp. primarily ingest roots and not soil, this might explain the only sporadic occurrence of N_2_O emissions of them. We don’t know of any study yet which has investigated N_2_O emissions from Scarabaeidae larvae. We have added this information to the discussion.

*Lines 274 to 282. I think this should be revised in line with all the suggestions and comments.*

REPLY: Please see our previous comments regarding that topic.

*Lines 289 to 307. Very good discussion.*

REPLY: Thank you.

*Lines 308 to 314 and S1_File. Why the authors think N2O flux is negative in a few occasions?*

REPLY: See previous reply.

*Line 310. Not “considerable,” but for ants there is some literature about N2O (Soper et al, 2019, etc.).*

REPLY: We have added that information to the manuscript.

*S1_File. The authors do not report the time of sampling, but I wonder if they would have found a diel pattern, for example, consistent with the soil CO2 diel pattern: when CO2 concentration in the soil is higher, do the larvae produce the same or less, since there is less O2?*

REPLY: We have included the sampling time in S1_File. Measurements were conducted between 9:00 and 15:00. This time span is not sufficient to detect any diurnal patterns, but it is definitely a very interesting question. Currently, we don’t even know if the larvae have a diurnal (vertical and horizontal) movement and feeding patterns in general. These question about larval ecology have to be answered first before we can take a closer look at diurnal variations in gas emissions.

*Grammar and standardization*

*I kindly suggest that the authors revise the manuscript for consistency in at least three points that I noticed:*

*1. The authors did not use the serial comma in some occasions, but they did in others. I suggest homogenizing the whole document using the serial comma. For example, in lines 22, 57, 58, 60, etc.*

REPLY: We checked the entire document to make sure we use the serial comma consistently.

*2. A comma should not separate the subject and the verb. For example, in line 20, I believe that the correct way to write this is "…from laboratory experiments is often controversial, and it has been limited...". In lines 48 and 49 “… (N2O), but they are also…”. Other examples of missing subject after comma are in lines 64, 65, 133, etc.*

REPLY: The examples you are listing have been checked by a native speaker and the use of commas is correct. However, we have to admit, as Germans we tend to construct complicated sentence structures.

*3. I suggest following the International System units and rules. A checklist can be found online (I use this one https://physics.nist.gov/cuu/Units/checklist.html). For example, in lines 31 and 32, units must be provided after the amounts, and I would use “Gg” (giga grams) instead of “kt” (unless the authors think those units are better for broader communication): “10.42 Gg CO2 yr-1 and 409.53 Gg CO2 yr 1, and 0.01 Gg CH4 yr-1 and 1.36 Gg CH4 yr-1.”. The same occurs in line 74, 88, 89, 99, 100, 149, 150, 163, etc. In line 104, there should be a space character in “1 m”. In line 109, I believe the authors want to use “×” (Alt + 0215) instead of “x”. In lines 127, 128, and 132, change “ml” to “mL”. Line 146, use “Pa” or “kPa” instead of “PSI”. And there may be other excerpts needing standardization that the authors will likely find quickly with the help of the checklist.*

REPLY: We are aware that we use units in our manuscript which are commonly used in our research field, but are not SI units. However, these are non-SI units which are accepted for use with the SI units. We would like to refer for this to the official SI brochure, table 8 (<https://www.bipm.org/utils/common/pdf/si-brochure/SI-Brochure-9-EN.pdf>).

The “x” and the space character have been corrected. We kept the PSI value because this is the unit shown by the instrument, but inserted kPa in brackets.

*References*

*Bollazzi M, Forti LC, Roces F (2012) Ventilation of the giant nests of Atta leaf-cutting ants: does underground circulating air enter the fungus chambers? Insect. Soc 59:487–498. https://doi.org/10.1007/s00040-012-0243-9*

*Kleineidam C, Roces F (2000) Carbon dioxide concentrations and nest ventilation in nests of the leaf-cutting ant Atta vollenweideri. Insect Soc 47:241–248. https://doi.org/10.1007/PL00001710*

*Kleineidam C, Ernst R, Roces F (2001) Wind-induced ventilation of the giant nests of the leaf-cutting ant Atta vollenweideri. Naturwis-senschaften 88:301–305. https://doi.org/10.1007/s001140100235*

*Fernandez-Bou, A. S., Dierick, D., Swanson, A. C., Allen, M. F., Alvarado, A. G. F., Artavia-León, A., et al. (2019). The role of the ecosystem engineer, the leaf-cutter ant Atta cephalotes, on soil CO2 dynamics in a wet tropical rainforest. Journal of Geophysical Research: Biogeosciences, 124, 260– 273. https://doi.org/10.1029/2018JG004723*

*Fernandez-Bou, A.S., Dierick, D. & Harmon, T.C. Oecologia (2020). Diel pattern driven by free convection controls leaf‑cutter ant nest ventilation and greenhouse gas emissions in a Neotropical rain forest. https://doi.org/10.1007/s00442-020-04602-2*

*Soper FM, Sullivan BW, Osborne BB et al (2019) Leaf-cutter ants engineer large nitrous oxide hot spots in tropical forests. Proc R Soc B Biol Sci 286:20182504. https://doi.org/10.1098/rspb.2018.2504*

*Ohashi M, Finér L, Domisch T et al (2005) CO2 efflux from a red wood ant mound in a boreal forest. Agric For Meteorol 130:131–136. https://doi.org/10.1016/j.agrformet.2005.03.002*

*Risch A, Schuetz M, Jurgensen MF et al (2005) CO2 emissions from red wood ant (Formica rufa group) mounds: seasonal and diurnal patterns related to air temperature. Ann Zool Fenn 42:283–290. https://www.jstor.org/stable/23735916*

REPLY: Thank you for these references.

**Point-by-point reply to Reviewer #2**

*This is an interesting topic, how Scarabaeidae larvae can affect GHG emissions from soils. These emissions by the soil animals are not well studied, and therefore this manuscript would increase our knowledge about the topic.*

REPLY: We would like to thank the reviewer for the evaluation of our manuscript and the constructive criticism. We hope we could dispel the reviewer’s concerns with our revision.

*However, I am concerned about few points.*

*1.* *It is not clear how the emissons from the larvae were actually calculated. One gas sample was taken about 1 hour (why not exactly the same time always?) after sealing the animal in a bottle and then ambient sample was taken (not described how) and then there was a blank? Empty bottle? Was the emisson calculated just by dividing the final concentration by time? (Measured concentrations are not given in the manuscript). If so this might give the wrong result or not totally comparable results if the closing time is different.*

REPLY: We have rephrased the respective part of the method section to make the description of the emission calculation more understandable. Due to logistical constraints (incubations and plot excavations had to be conducted in parallel), it was not possible to finish each incubation after exactly one hour. However, we did not observe any correlation between incubation duration and emission strength (see S1_File). The blank was indeed just an empty glass vial for testing potential effects by the used rubber stopper on the measurement results. We have added a description of how the ambient sample was taken. Measured concentrations were added to the manuscript as well as to the supplemental material.

*2. When the larvae were taken out from their natural environment they might be stressed and it would affect perhaps their behaviour. This could explain differences between measurements in the field and in the laboratory. BUT I would not call the measurements in the field "Field measurements" because the animals are taken out from their natural habitat.*

REPLY: We agree that differences between field and laboratory measurements could be the result of a larval stress reaction. We added this to the discussion. However, we disagree with the notion not to describe the field incubations as such. The applied method is not a gold standard field method, i.e. it does not provide a non-invasive estimate of the larval greenhouse gas emissions. There is no established method yet to retrieve such an estimate. The method applied in this manuscript is the closest we can get to such an estimate currently. Does it introduce a bias? Certainly, but we don’t know yet how significant this bias is and this is a point which has to be discussed. But calling a measurement not a field measurement because an organism is taken out of its natural habitat for the actual measurement, or the natural habitat is disturbed by the measurement, would declassify a whole range of ecological measurement methods as field methods. We totally agree that the method we employed is an imperfect field measurement method, but nevertheless, we still regard our results as field measurements.

*3. Based on 1 and 2 I would be very careful in upscaling the results, also the small number of sites and one sampling time and lack of real field measurements are affecting the quality of the data.*

REPLY: We share the concerns raised by the reviewer and rephrased the respective parts of the manuscript to make this clear. We hope, it is now clear that the upscaling exercise is really simple and it just serves the purpose to put Scarabaeidae larvae ‘on the map’ of the research community as potential greenhouse gas sources warranting more research. We were really conservative in our approach by restricting the upscaling exercise only to the species we actually investigated.

*Some minor comments:*

*-were the larvae cleaned /washed from soil before measurements...? Or measured with soil?*

REPLY: No, the larvae were not cleaned as it was not necessary. We observed that apparently healthy larvae are not dirty when excavated. Soil particles do not stick to them. So, there were no soil particles with the larvae in the glass tubes (unless they defecated during the incubation). We added that to the text.

*-Line 134 Why carrot was given, do they eat carrot in nature?*

REPLY: These larvae are not very picky when it comes to choosing roots for eating, but they really seem to love carrots. Based on previous experience, carrots seem to be the best fodder to keep the larvae thriving in captivity. We managed to keep larvae alive for years and to get them to pupate successfully in the laboratory just on carrots. For this study, we tried to provide them with the most optimal laboratory conditions for growth to counterbalance potential stress reactions.

*-Line 135 tap water (not tab)*

REPLY: Corrected.

*-Line 149-150 calibration was done with very high concentrations, you did not show the concentrations measured from the bottles, were they in the range of standards?*

REPLY: Actually, the opposite turned out to be true. For a range of samples, it would have been better to have gas standards with even higher concentrations available. We added information on the gas concentrations in the samples to the manuscript and the supplemental material.

*-Is Fig 2 repeating the same data as in Fig 1?*

REPLY: Both figures use the same data, but the data treatments differ between the two figures to highlight different aspects of the dataset.

*-Supplement Figure1, This figure is quite complicated to read, It does not open to me, could you split the data into several tables/figures. Units are missing from the scales!*

REPLY: This is already a simplified figure and a reduced version of the original one. We cannot simplify it any further, but we added a description of how to read this figure for those unfamiliar with this display type. We can assure you that there are no units missing, which becomes hopefully clear through the added description. Units missing at a first glance are just positioned at the opposite site of the figure to avoid overlapping of adjacent scales.
