# Supplementary material for: First field estimation of greenhouse gas release from European soil-dwelling Scarabaeidae larvae targeting the genus Melolontha
Source: PLoS One. 2020 Aug 26;15(8):e0238057. doi: 10.1371/journal.pone.0238057 (PMC7449402; doi:10.1371/journal.pone.0238057)
Supplement: Attachment — Submitted filename: Response to Reviewers.docx [file pone.0238057.s007.docx]

**Author Reply**

**First field estimation of greenhouse gas emissions from European soil-dwelling Scarabaeidae larvae targeting the genus *Melolontha***

Authors: Carolyn-Monika Görres^1,2,*^, Claudia Kammann^1^

^1^ Department of Applied Ecology, Hochschule Geisenheim University, Geisenheim, Germany

^2^ Department of Soil Science and Plant Nutrition, Hochschule Geisenheim University, Geisenheim, Germany

**Point-by-point reply to Reviewer #1**

*Hello again, Dr. Görres and Dr. Kammann. I hope you are doing well.*

*I think you have done a very nice job responding to the reviewers' questions and addressing the comments. I also appreciate your clarifications across the manuscript thinking about a broader audience.*

*In my opinion, your manuscript is ready for publication.*

*Best luck on your future research.*

*Angel S.*

REPLY: Dear Angel, thank you very much for taking the time to review our revised manuscript and for your kind words. We really appreciated your constructive comments for the revision and we are glad that we could dispel your concerns about the manuscript and address your comments in a satisfactory manner.

**Point-by-point reply to Reviewer #3**

*The manuscript is well written and has concise, well connected sentences. In this study, the authors have made an attempt to do field and lab scale measurement of greenhouse gas emissions from Scarabaeidae larvae. I really liked the idea of upscaling the results in order to provide better understanding of the impacts of such emissions. Several studies talk about microbial decomposition of soil organic carbon and how that influences the atmospheric greenhouse gas concentrations but very few have talked about other faunal groups such as ants, termites and beetle larva. So, this is a good attempt to quantify that. I have gone through the responses to reviewers’ comments and most of them seem satisfactory to me. Some of my overall concerns on the methodology of this manuscript are below.*

REPLY: We would like to thank the reviewer for taking the time to review our revised manuscript. We highly appreciate the constructive criticism and the kind comments.

*The larvae were incubated at the soil surface in field, but the larvae were actually excavated from deeper depths. Do you think by doing so, we are adding a bias to the greenhouse gas emissions that actually comes from larvae at deeper depths versus what is reported in the manuscript?*

REPLY: Yes, it is highly likely that this approach introduced some kind of bias in the measured greenhouse gas emissions. At the moment, we do not know the magnitude of this bias and if it increased or decreased emissions because there are several (potentially) co-occurring processes that might either cancel each other out or amplify each other. Larvae can release gases via their respiratory system (i.e. tracheal tubes and spiracles) and/or via their anus. For CO_2_ it is certainly a mixture of both pathways, whereas gases produced in the digestive tract like CH_4_ and N_2_O probably are released primarily via the anus. But an unknown proportion of these gases could be released via the respiratory system as well. The gas volume released via the respiratory system depends to a large degree on the metabolic rate which in turn is affected by temperature and (feeding) activity. So incubating larvae at the soil surface at higher temperatures might have increased their respiration. However, strong changes in air oxygen as well as air moisture content might have affected larval respiration activity in a negative way. For the anus pathway, the question is whether the excavation itself stresses the animals to such a degree that they already release a big gas plume before they are sealed inside the incubation vessel. In that case, we might underestimate larval greenhouse gas emissions.

We added these thoughts to the discussion. For the future, we are planning various tests to assess in detail the bias in larval greenhouse gas emission estimates introduced by this sampling method.

*How the calculations for of these emissions were done?*

REPLY: We added the detailed emission calculations to S1 File in the supporting information.

*Also, the larvae used in field study and lab incubation, were they all similar in size? If not, do you think that would influence the greenhouse gas emissions as well?*

REPLY: We assume that with size you are referring to the dimensions of the larval bodies, not larval biomass. We do not have any size measurements. We could have used larval length and/or head capsule width, but they were much more difficult to measure (and less precise) than simply larval biomass due to the high activity of these cockchafer larvae. And they always try to keep their body in a C shape. Thus, we relied solely on larval biomass measurements and in that regard, the groups did not differ significantly. From a visual impression, they were all similar in size. In general, differences in size would most certainly influence greenhouse gas emissions and they should also translate into larval biomass differences.

*Other than these minor concerns, I have gone through the responses to the reviewers and they seem convincing and satisfactory to me.*

REPLY: Thank you again for your comments and we hope, we could satisfactorily address your concerns.

**Additional comments to the editor**

In addition to the reviewers’ comments, we have found two other points in the manuscript that we thought needed revision.

First, we corrected an error in one of the y-axis labels of Figure 3. For the CH_4_ emissions (top panel), we replaced “µg h^-1^ m^-2^” with “µg h^-1^ larva^-1^”.

Second, at the end of the discussion we originally stated that there is no literature available on N_2_O emissions from soil-dwelling beetle larvae. Recently, we discovered that this is not correct. We found a publication from 2014 which quantified N_2_O emissions of white grubs and have included this paper in the discussion.
